# Using automated electronic medical record data extraction to model ALS survival and progression

**DOI:** 10.1186/s12883-018-1208-z

**Published:** 2018-12-14

**Authors:** Alex G. Karanevich, Luke J. Weisbrod, Omar Jawdat, Richard J. Barohn, Byron J. Gajewski, Jianghua He, Jeffrey M. Statland

**Affiliations:** 1 Department of Biostatistics, University of Kansas Medical Center, Kansas City, USA; 2 School of Medicine, University of Kansas Medical Center, Kansas City, USA; 3 Department of Neurology, University of Kansas Medical Center, Kansas City, USA; 4 EMB Statistical Solutions, LLC, Overland Park, KS 66210 USA

**Keywords:** Amyotrophic lateral sclerosis, Motor neuron disease, Disease progression, Electronic medical record

## Abstract

**Background:**

To assess the feasibility of using automated capture of Electronic Medical Record (EMR) data to build predictive models for amyotrophic lateral sclerosis (ALS) outcomes.

**Methods:**

We used an Informatics for Integrating Biology and the Bedside search discovery tool to identify and extract data from 354 ALS patients from the University of Kansas Medical Center EMR. The completeness and integrity of the data extraction were verified by manual chart review. A linear mixed model was used to model disease progression. Cox proportional hazards models were used to investigate the effects of BMI, gender, and age on survival.

**Results:**

Data extracted from the EMR was sufficient to create simple models of disease progression and survival. Several key variables of interest were unavailable without including a manual chart review. The average ALS Functional Rating Scale – Revised (ALSFRS-R) baseline score at first clinical visit was 34.08, and average decline was − 0.64 per month. Median survival was 27 months after first visit. Higher baseline ALSFRS-R score and BMI were associated with improved survival, higher baseline age was associated with decreased survival.

**Conclusions:**

This study serves to show that EMR-captured data can be extracted and used to track outcomes in an ALS clinic setting, potentially important for post-marketing research of new drugs, or as historical controls for future studies. However, as automated EMR-based data extraction becomes more widely used there will be a need to standardize ALS data elements and clinical forms for data capture so data can be pooled across academic centers.

**Electronic supplementary material:**

The online version of this article (10.1186/s12883-018-1208-z) contains supplementary material, which is available to authorized users.

## Background

Amyotrophic Lateral Sclerosis (ALS) is a fatal neuro-degenerative disease. While over 50 clinical trials have been conducted over the last two decades, none have been successful save riluzole and edaravone [1], which at best offer modest improvements in survival or function [2]. While many studies may have failed because the drugs were ineffective, a recurring theme in ALS are trials which do not meet their primary outcome but yield indeterminate results [3]. Two major hurdles to conducting ALS trials are the rarity of ALS (3.9 in every 100,000 people in the US [4]) and the disease’s heterogeneity [5], which is a barrier to properly powered studies.

Methodology for rare-disease clinical trials is an important area of study for ALS researchers [6]. Enriching trials with historic controls has become possible due to the creation of large pooled placebo data sets [7] and is an approach used for selection of drugs for larger studies, such as in the lithium and rasagiline study [8–10]. Other benefits to large databases of ALS patients include constructing predictive models for screening particular subgroups of patients, which could reduce the heterogeneity of disease progression observed in the trial, or making interim decisions during the conduct of a clinical trial based on predicted and observed disease progression.

The wide implementation of Electronic Medical Record (EMR) systems across the United States, using one of two commercial systems, and the development automated abstraction and de-identification of data, create opportunities to: 1) better understand ALS disease progression and determinants of survival in the clinical setting; 2) use clinical data to enrich existing placebo-arm data sets to improve the power of trials; and 3) leverage this electronic infrastructure to run clinical trials – including EMR-based screening, randomization, and data collection. For these approaches to be worthwhile, we need to be able to demonstrate the feasibility of automatically extracting the data required for modelling ALS disease progression and survival directly from the EMR.

We consider the feasibility of constructing statistical models built with automatically captured EMR patient data from our ALS clinic at the University of Kansas Medical Center (KUMC). This is a key first-step in utilizing the EMR to augment clinical trials.

## Methods

### Study design

We first determined what specific data was necessary to build models for ALS disease progression and survival. Variables of interest for such models include, at a minimum, demographic information (age, race, and gender), survival information (vital status and date of death), ages of disease onset and diagnosis [5, 11], site of disease onset (typically bulbar or limb) [5, 12–14], riluzole use, BMI [5, 15], FVC [15, 16], and ALS Functional Rating Scale – Revised (ALSFRS-R) score [13, 17–19]. The ALSFRS-R, which is the gold-standard for measuring ALS disease progression, is a clinician-administered series of twelve questions which concern the ability to perform basic functional activities such as eating, walking, dressing, and breathing. Each question is rated on a 0–4 scale, with the overall score of 48 representing normal function [20].

To determine if these variables could be automatically extracted from the EMR, we conducted a retrospective chart review of patients seen at the KUMC ALS Clinic between summer 2013 and summer 2016. We obtained this data directly from the EMR using the KUMC Healthcare Enterprise Repository for Ontological Narration (HERON), powered by Informatics for Integrating Biology and the Bedside (i2b2), a discovery tool that allows searches of de-identified EMR data [21–23]. KUMC’s EMR is provided by Epic (EPIC EMR system, Epic Systems Corporation, Verona, USA, 2015. Using patient’s medical record numbers, this dataset was then verified for completeness and accuracy by manual review of the EMR records. Because we were interested in considering the efficiency of using automated tools versus manual review, the number of hours spent performing the automated review and manual review were tracked.

### Study population

**Fig. 1:**
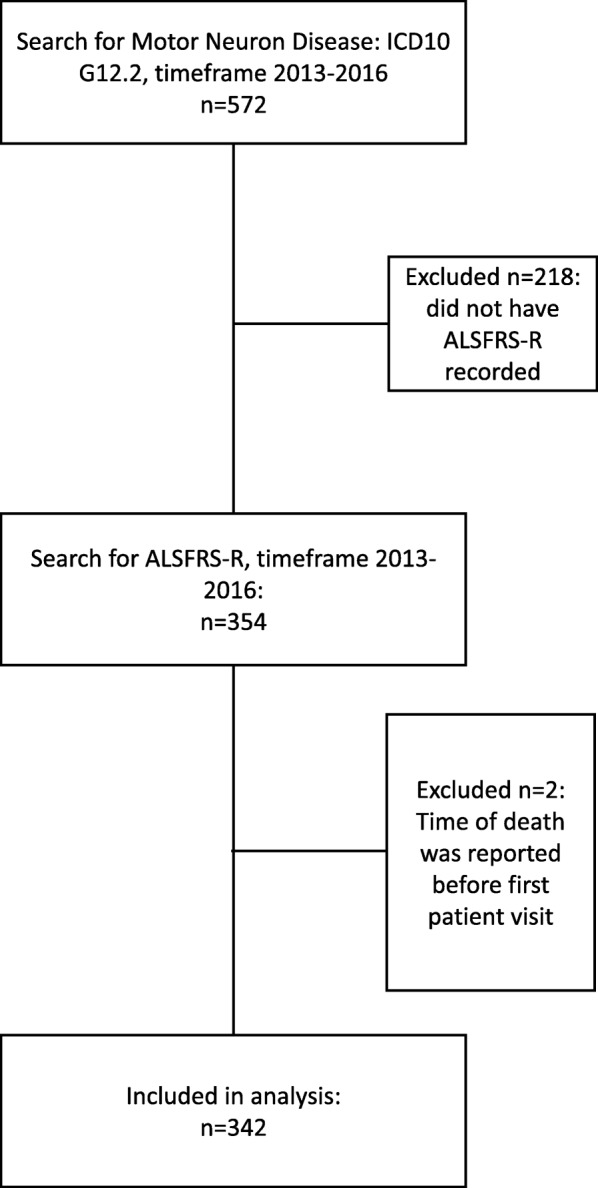
Patient inclusion / exclusion in study

The ALS clinic at the University of Kansas Medical Center (KUMC) serves roughly 4 state regions across the Midwest (Kansas, Missouri, Oklahoma, Arkansas). At each visit, patient data collected by the clinician is entered in the EMR. Using HERON, we first performed a search using the ICD10 code for motor neuron disease and at least one visit. This would represent the full pool of patients seen in clinic over this time frame. Next we reduced this to patients having at least one ALSFRS-R score entered into the EMR (Fig. 1). Only patients seen in the ALS specialty clinic with a known diagnosis of motor neuron disease have ALSFRS-R scores in the EMR.

### Statistical methods

#### Analysis of disease progression

Disease progression is measured by patients’ average change per month in ALSFRS-R score. Each patient’s disease progression vs. time (as months since first clinical visit, where the first clinical visit is time 0) was modelled via a linear mixed model which included random slopes and random intercepts (these were allowed to correlate); the fixed effects for the intercept and slope of this model represent the average baseline ALSFRS-R score and average change in ALSFRS-R per month for the clinic. Individual estimates of patient baseline ALSFRS-R score can be obtained by adding the estimated fixed intercept effect to the patient’s estimated random intercept effect; similarly the individual estimate of a patient’s change in ALSFRS-R per month can be estimated by adding the fixed slope effect to the patient’s estimated random slope effect. Linearity was assumed from the literature [14, 24, 25] and verified via diagnostic plots (Additional file 1). These models were fit using the nlme package in R [26].

#### Analysis of survival

Our survival model analyzed time from patients’ first clinical visit to death (or censoring). Survival data captured by HERON includes both data from the EMR and from the Social Security Death Index [27]. Median survival was estimated by via a Kaplan-Meier approach with interval given by the log-log transformation. A Cox Proportional Hazards model was employed to assess the simultaneous effects of available predictors: BMI, age, and ALSFRS-R score at first visit, and gender. 72 patients were missing baseline BMI scores and were excluded from the Cox model. All analyses were done using R (version 3.2.4) [28].

## Results

### Accuracy of EMR data

A general search based on ICD10 code identified 572 subjects; 354 patients had at least one ALSFRS-R recorded in the EMR (62.4%), 352 of which were deemed eligible for analysis (two were excluded due to nonsensical death dates) (Fig. 1). Manual review verified ALSFRS-R and sub-scores as accurate.

Many variables of interest for modeling progression and survival (time of disease onset, time of disease diagnosis, and site of disease onset) were only available by manual chart review, because the EMR did not yet have a dedicated field to capture such information (Table 1). Other variables, though extracted via HERON, were not useful for analysis due to extreme sparsity (for example, raw FVC was missing from 59% of records).

**Table 1:** Specific data automatically extracted from KUMC EMR by HERON, and data that required a manual chart review

	Information automatically extracted from the EMR using HERON	Information requiring manual chart review
Demographic data	Subject age, race, gender, ethnicity	Date of disease onset, date of diagnosis, site of disease onset
Longitudinal data	ALSFRS-R and its sub scores, BMI, FVC (raw and percent-predicted)	
Medication history		Riluzole use
Survival data	Death status, date of death	

The time spent coordinating with the team at HERON to properly identify and extract variables of interest took roughly 3 h. The manual review took over 30 h. Once the variables of interest were properly identified within the EMR, obtaining the data through HERON became a matter of minutes rather than hours.

### Patient characteristics

Table 2 reports patient characteristics: participants at KUMC were predominantly male (57%), had an average age at first clinical visit of 64.1 years, 65% with limb onset, 63% taking riluzole, with an average ALSFRS-R at first visit of 34.5.

**Table 2:** Demographic information of KUMC ALS clinical patients. Baseline is defined as the time of a patient’s first recorded ALSFRS-R score at KUMC

	KUMC
Number of patients	352
Percent female / Male	43/ 57
Percent Caucasian / Non-Caucasian	89/ 11
Percent limb onset / Bulbar / Other	65/ 27/ 8
Percent using riluzole Yes / No / NA	63/ 35 / 2
Percent survived during follow up	69
Median time from baseline to last record, months (IQR)	7.1 (17.7)
Median age at baseline (IQR)	65.3 (11.2)
Median number of months from onset to baseline (IQR)	19.0 (28.1)
Median baseline FVC percent predicted (IQR)	70.0 (32.5)
Median BMI at baseline (IQR)	26.4 (7.23)
Median number of ALSFRS-R assessments (IQR)	3 (4)
Median baseline ALSFRS-R (SD)	35.5 (10.0)

### Statistical results

#### Analysis of disease progression

**Fig. 2:**
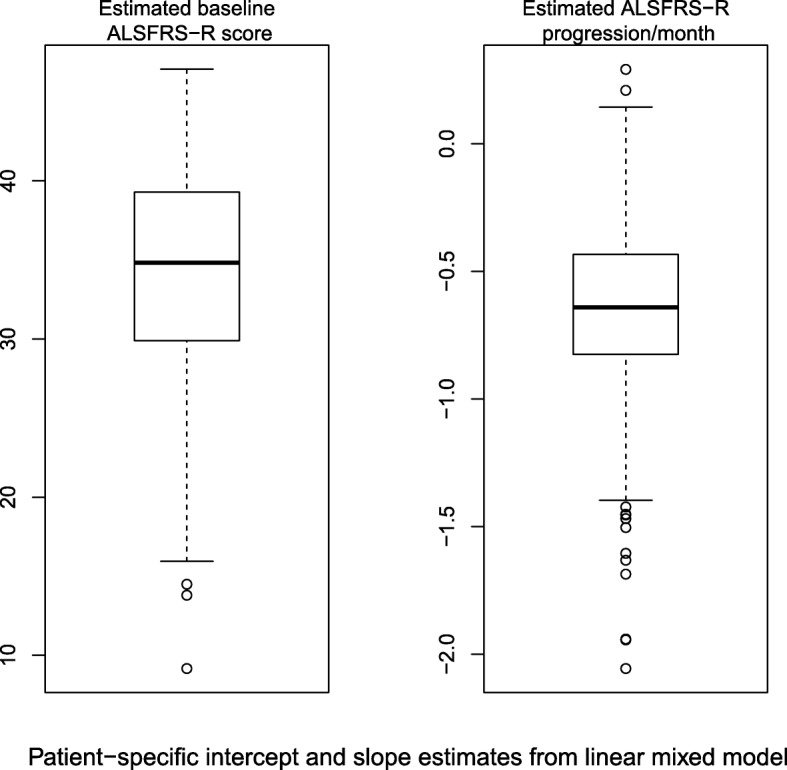
The distributions of baseline scores and monthly disease progression as estimated by the linear mixed effects model. These were calculated from the model by adding the fixed effect of intercept (or slope) to each patient’s estimated random effect of intercept (or slope)

The fixed-effect of clinic-level baseline ALSFRS-R score was 34.08 with 95% interval (33.28, 34.88), with random-effect standard deviation of 7.08 with 95% interval (6.49, 7.28). The fixed-effect of clinic-level disease progression (in terms of loss of ALSFRS-R per month) was 0.64 with 95% interval (0.56, 0.73), with random-effect standard deviation of 0.56 with 95% interval (0.48, 0.65). See Fig. 2 for graphical representation of the estimates of disease progression and baseline ALSFRS-R score by patient.

#### Analysis of survival

**Fig. 3:**
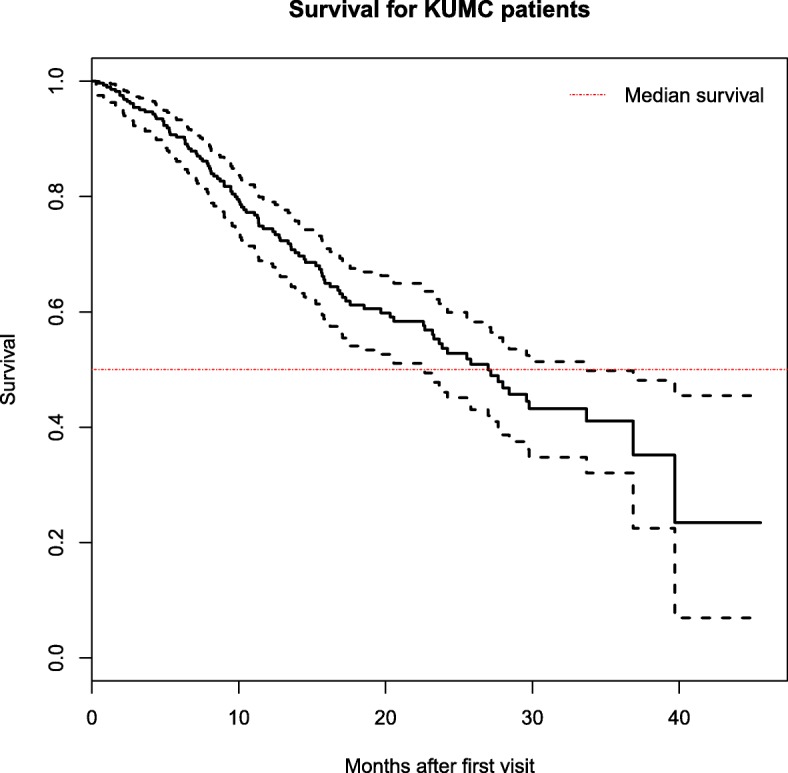
Kaplan-Meier survival curves and 95% confidence interval for KUMC patients. The horizontal line represents the median survival time, equivalent to where the y-axis is 0.5

Median survival time from first visit was 27 months (95% interval (22.7, 33.7)) for KUMC patients, as per Kaplan-Meier model. The Kaplan-Meier survival curve (unadjusted for other covariates) is given in Fig. 3. We observed a large number of censored observations (69% censored).

Our Cox proportional hazards model found baseline ALSFRS-R score, baseline age, and baseline BMI as significant (*p* < 0.05) predictors of survival when *α* = 0.05. Higher baseline ALSFRS-R and BMI were related with improved survival, while higher baseline age was associated with decreased survival (Table 3).

**Table 3:** Hazard ratios from Cox model

Survival Model			
Covariate	Hazard Ratio	95% CI	*P*-value
Age (at onset)	1.025	(1.004, 1.047)	0.019
Sex (male)	0.732	(0.473, 1.133)	0.161
BMI (baseline)	0.950	(0.909, 0.993)	0.022
ALSFRS-R total (baseline)	0.939	(0.909, 0.970)	<0.001

Hazard ratios below one result in improved survival

## Discussion

Here we demonstrate the feasibility of using an automated extraction tool (HERON) to obtain ALS patient data directly from the KUMC EMR which could be used for analysis of ALS disease progression and survival. While data pertaining to demographic, ALSFRS-R, and survival information was both readily obtainable and accurate, some key variables (especially disease onset time and riluzole use) were only available via manual EMR review and/or suffered from large amounts of missing data.

The main advantages to using automatic tools such as HERON includes that they can drastically reduce the amount of time needed to accurately capture EMR data when compared to a manual review of the EMR. This methodology is generalizable across other research sites: EPIC is one of the two major EMR record systems in the US, serving over 50% of patients in the US [29], and represents a large number of academic centers with ALS clinics. The automatic extraction tool HERON is powered by i2b2, which is used by dozens of research institutions within the US and abroad [30].

Looking towards the future, as EMR data becomes more complete, other advantages of using this approach will emerge. Advantages to complete and comprehensive ALS records in the EMR include allowing clinicians to track the performance of their patients clinic-wide and compare these to other ALS clinics, for both research and quality control purposes. For example, the average ALSFRS-R decline per month in the KUMC clinic of 0.64 is somewhat high compared to reports from other clinics, which report monthly ALSFRS-R declines of between 0.36 to 0.65 [14, 31–33]. Note that this may be because we were unable to adjust for how long patients’ have had the disease.

Other future advantages include the ability to perform retrospective studies quickly and efficiently, which could create support for new therapeutics or improvements to standards of care. This depends heavily on tracking of patients’ use of therapeutics in a way that is accessible in the EMR. EMR data could also be used to augment clinical trial data, being used as either a placebo/ standard of care arm or as historical controls [34]. This has become a vital issue for the broader ALS community. For example, approval of edaravone in the US has raised many questions about which patients will benefit from this therapy and for how long. This could be answered by pooling ALS clinic data. In addition, edaravone has put a limit on how broadly existing placebo data sets like PRO-ACT can be used for historical controls in clinical trials. Contemporary controls captured through automated EMR data abstraction could be one solution to this problem [1, 35, 36].

One current criticism of ALS clinical trials is that the ALS patients who serve in these trials are not representative of the general population [37], which is likely due to the rigorous inclusion/exclusion criteria for these trials. One simple solution to make ALS trials more representative is to simply modify the inclusion/exclusion criteria – however the resulting increased patient variability would require very large studies. Again we see the potential utility of EMR data: with a more general trial population, we would be free to use the EMR to augment the control population for these trials. Networks such as the Northeast or Western ALS Study Groups [38] could provide placebo or standard-of-care arms in a variety of designs, and could make such large-scale studies possible.

The main disadvantage of this approach is the current lack of completeness of the EMR with respect to critical ALS data, resulting in incomplete statistical models. To use the EMR as we propose across multiple academic centers, the ALS community would need to agree on a set of common data elements or ALS-related forms to capture in the EMR. Such agreement could allow common data dictionaries to be used to allow for automated data capture not just across academic centers, but across different EMR platforms (i.e. Epic and Cerner). Furthermore, physicians and their clinic personnel would need to adhere to these data dictionaries, and then rigorously enter all the required data for each patient at each visit. Many efforts have already been made toward developing these common data sets for ALS: much of the field already captures the ALSFRS-R, the FVC, and details about the diagnosis at each visit. In addition several initiatives are underway to standardize forms across institutions, with a suite of ALS clinic forms available for download through Epic Central.

One example of critical information that needs to be collected in a standardized way is disease onset time. Because disease duration (which is derived from disease onset time) is critical for both survival and disease progression modelling [5, 12, 24, 25, 39], it is necessary that ALS clinics dedicate a data-capture form for this, as opposed to entering it as free-text notes/comments where it is difficult to find systematically. Other critical variables include usage of approved therapeutics (such as riluzole or ederavone), time of diagnosis, and location of symptom onset.

## Conclusions

We were able to use automated extraction tools to accurately obtain necessary variables from the EMR with which to create simple statistical models of both ALS disease progression and survival time. Key variables that might offer large improvements to these models (such as disease onset time or riluzole use) were unavailable via automatic extraction. In the future, as automated EMR data abstraction becomes increasingly important for post-marketing surveillance of FDA approved drugs, or for use as concurrent controls, the ALS community will need to adopt common data elements for the EMR. Optimal use of the EMR requires disease-specific key variables, such as disease-onset time for ALS, to be identifiable and obtainable by data extraction tools as well as rigorous data entry by clinical staff.

## Additional file

Additional file 1: Linearity of 16 randomly selected patients who had > 3 visits. For 16 randomly selected patients with more than three recorded visits, we show their ALSFRS-R score versus time in months, along with the fit regression line. This gives the reader a general idea of the linear decline of the ALSFRS-R seen in patients. (PDF 8 kb)

## Abbreviations

ALS: Amyotrophic lateral sclerosis
ALSFRS-R: Amyotrophic lateral sclerosis functional rating scale – revised
EMR: Electronic medical record
FVC: Forced vital capacity
HERON: Healthcare Enterprise Repository for Ontological Narration
I2b2: Informatics for Integrating Biology and the Bedside
KUMC: University of Kansas Medical Center
