# Supplementary figures and images for: Using automated electronic medical record data extraction to model ALS survival and progression
Source: BMC Neurol. 2018 Dec 14;18:205. doi: 10.1186/s12883-018-1208-z (PMC6295028; doi:10.1186/s12883-018-1208-z)

# Linearity of 16 randomly selected patients who had > 3 visits

ALSFRS-R Score

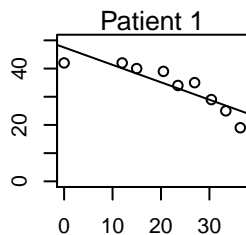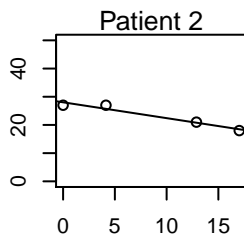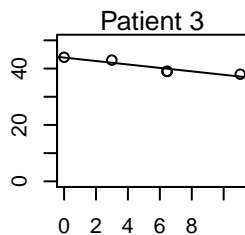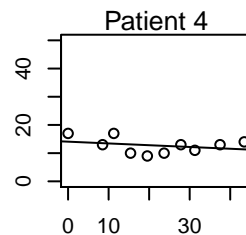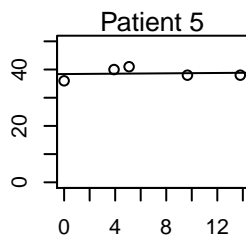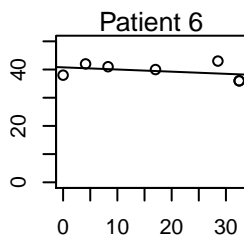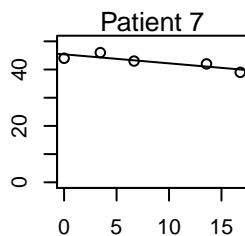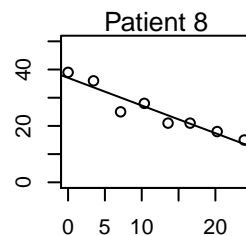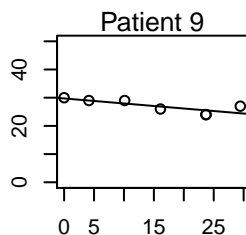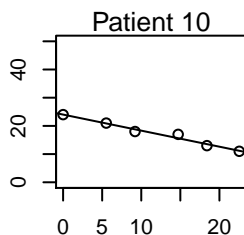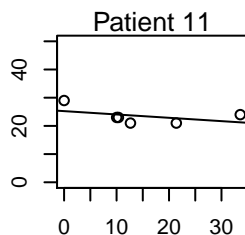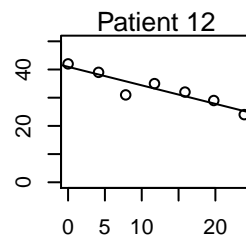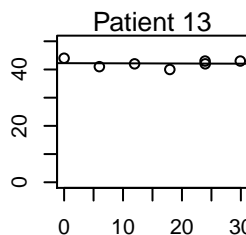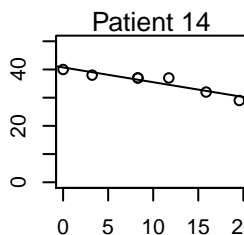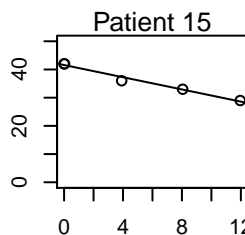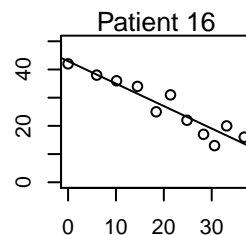

Time (months)

Supplement: Supplementary file 1 — Linearity of 16 randomly selected patients who had > 3 visits. For 16 randomly selected patients with more than three recorded visits, we show their ALSFRS-R score versus time in months, along with the fit regression line. This gives the reader a general idea of the linear decline of the ALSFRS-R seen in patients. (PDF 8 kb) [file 12883_2018_1208_MOESM1_ESM.pdf]
